# The *Caenorhabditis elegans* homolog of human copper chaperone Atox1, CUC-1, aids in distal tip cell migration

**DOI:** 10.1007/s10534-020-00239-z

**Published:** 2020-06-06

**Authors:** Xiaolu Zhang, Stéphanie Blockhuys, Ranjan Devkota, Marc Pilon, Pernilla Wittung-Stafshede

**Affiliations:** 1 Department of Biology and Biological Engineering, Chalmers University of Technology, 412 96 Gothenburg, Sweden; 2 Department of Chemistry and Molecular Biology, University of Gothenburg, 41390 Gothenburg, Sweden

**Keywords:** Cell migration, Distal tip cell migration, Atox1, CUC-1, *Caenorhabditis elegans*, Copper transport

## Abstract

Cell migration is a fundamental biological process involved in for example embryonic development, immune system and wound healing. Cell migration is also a key step in cancer metastasis and the human copper chaperone Atox1 was recently found to facilitate this process in breast cancer cells. To explore the role of the copper chaperone in other cell migration processes, we here investigated the putative involvement of an Atox1 homolog in *Caenorhabditis elegans*, CUC-1, in distal tip cell migration, which is a key process during the development of the *C. elegans* gonad. Using knock-out worms, in which the *cuc-1* gene was removed by CRISPR-Cas9 technology, we probed life span, brood size, as well as distal tip cell migration in the absence or presence of supplemented copper. Upon scoring of gonads, we found that *cuc-1* knock-out, but not wild-type, worms exhibited distal tip cell migration defects in approximately 10–15% of animals and, had a significantly reduced brood size. Importantly, the distal tip cell migration defect was rescued by a wild-type *cuc-1* transgene provided to *cuc-1* knock-out worms. The results obtained here for *C. elegans* CUC-1 imply that Atox1 homologs, in addition to their well-known cytoplasmic copper transport, may contribute to developmental cell migration processes.

## Introduction

Copper (Cu) is an essential nutrient in living organisms acting as a cofactor in proteins facilitating for example respiration, iron transport, oxidative stress protection, peptide hormone production, pigmentation, and blood clotting (Puig and Thiele 2002; Matson Dzebo et al. 2016). In order to avoid toxicity of free Cu ions, intracellular Cu is regulated by devoted Cu transport proteins that assist uptake, efflux, and distribution of the metal ion to load Cu-dependent proteins (Festa and Thiele 2011; Matson Dzebo et al. 2016). In human cells, after copper transporter protein 1 (Ctr1)-mediated uptake, the cytoplasmic Cu chaperone Atox1 transports Cu to ATP7A and ATP7B in the trans-Golgi network (Puig and Thiele 2002). In support of possible redundancy, it was recently shown that glutaredoxin 1 could replace Atox1 and deliver Cu to ATP7B (Maghool et al. 2020). ATP7A/B are P_1B_-type ATPases that use ATP hydrolysis to transfer Cu to the lumen for loading of target Cu-dependent enzymes, such as ceruloplasmin, tyrosinase and lysyl oxidase (Matson Dzebo et al. 2016). The Atox1-ATP7A/B Cu transport pathway is conserved in many organisms, including bacteria, yeast and (of importance for this study) the round-worm *Caenorhabditis elegans* (*C. elegans*) (Klomp et al. 1997; Wakabayashi et al. 1998, Puig and Thiele 2002).

Because Cu is important as a cofactor in many proteins enabling key biological processes (Turski and Thiele 2009; Grubman and White 2014; Matson Dzebo et al. 2016), it is no surprise that Cu is required in several distinguishing cancer phenomena, and cancer patients’ serum and tumors have increased Cu levels (Denoyer et al. 2015). We recently showed that Atox1 facilitates breast cancer cell migration (Blockhuys et al. 2020) and, upon analyzing breast cancer patient tumor data, high Atox1 levels in the tumors correlated with worse prognosis of patient survival (Blockhuys et al. 2020). In addition, we found Atox1 to localize to membrane protrusions in migrating breast cancer cells (Blockhuys and Wittung-Stafshede 2017), further supporting a role for Atox1 in cell migration. Most cancer patients die from complications of metastases (i.e., spreading of the primary tumor to other organs). Since a fundamental step in metastasis is cell migration, better understanding of cell migration mechanisms and pathways are of high importance.

Cancer and organism development may share many aspects (Bernhardt et al. 2012); for example, there is also a need for Cu during pregnancy such that even temporal nutritional Cu deficiency can result in long-lasting neurological effects on the offspring (Hamza et al. 2001; Keen et al. 2003). Moreover, mice in which *Atox1* was knocked out often died after birth, suggesting that Atox1 (and, thus, Cu transport) is important for appropriate embryo development (Hamza et al. 2001). When we probed *Atox1* expression during early (pre-implantation) mouse embryo development, we found its expression level to increase dramatically already at the eight-cell stage and, silencing of *Atox1* strongly diminished *Oct4* expression in mouse embryonic cells (Celauro et al. 2017). The Oct4 protein is a master transcription factor that regulates pluripotency and differentiation; notably, it controls an extensive gene network that drives the maternal-to-embryo transition (Zuccotti et al. 2011).

**Fig. 1:**
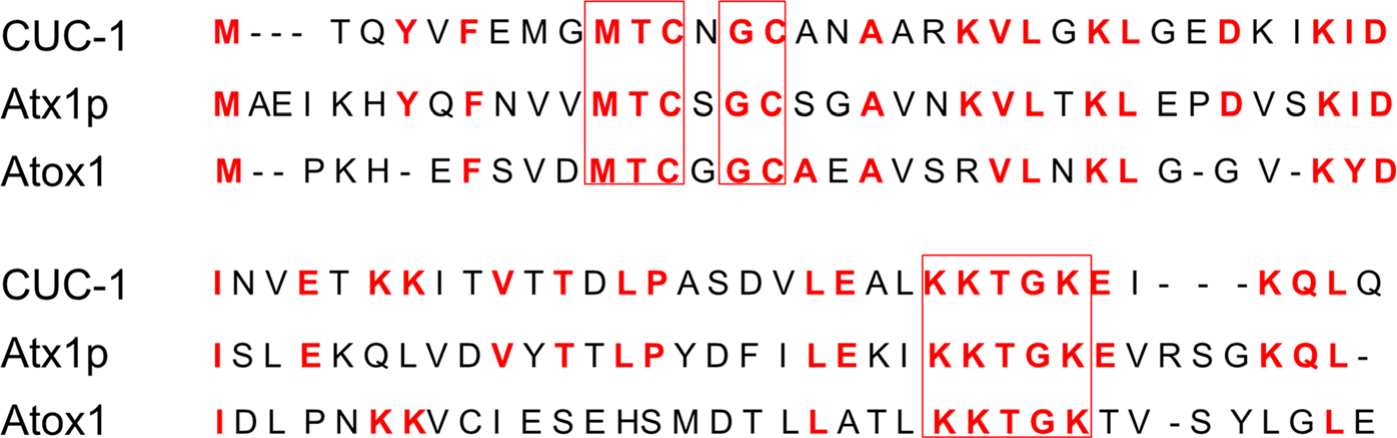
Amino acid sequence alignment of Atox1 homologs. The full amino-acid sequence of CUC-1 was aligned with full sequences of yeast Atx1p and human Atox1. Boxed residues are conserved in all three proteins. Residues in red are identical to CUC-1 in one or both other proteins. The top two boxes include the conserved MTCXGC Cu-binding motif (with the two cysteines coordinating the Cu ion)

To probe the hypothesis that Atox1, and its homologs, may play yet unexplored roles in developmental cell migration processes, we here turned to the model organism *C. elegans*. This is an excellent model system where at least 40% of human genes are conserved (Jorgensen and Mango 2002; Rual et al. 2004); moreover, the animals are easy to grow, have short life span and are transparent. The latter property allows for observation of individual cells and subcellular details during animal development using differential interference contrast (DIC) microscopy. Homologs to Atox1 and ATP7A/B in worms have been identified, named CUC-1 and CUA-1, respectively, and have demonstrated Cu transport functions (Wakabayashi et al. 1998; Chun et al. 2017). Notably, CUA-1 exhibits Cu-dependent redistribution in cells, similar to ATP7A/B in humans (Chun et al. 2017). CUC-1 has 39.1% sequence identity to Atox1 (Fig. 1) and could replace yeast Atx1 as a Cu chaperone in Atx1-deficient yeasts (Wakabayashi et al. 1998). However, in contrast to humans, where Atox1 is expressed ubiquitously, *C. elegans* CUC-1 showed tissue-specific expression, specifically found in intestinal cells of adult worms and hypodermal cells in the larvae (Wakabayashi et al. 1998; Chun et al. 2017).

**Fig. 2:**
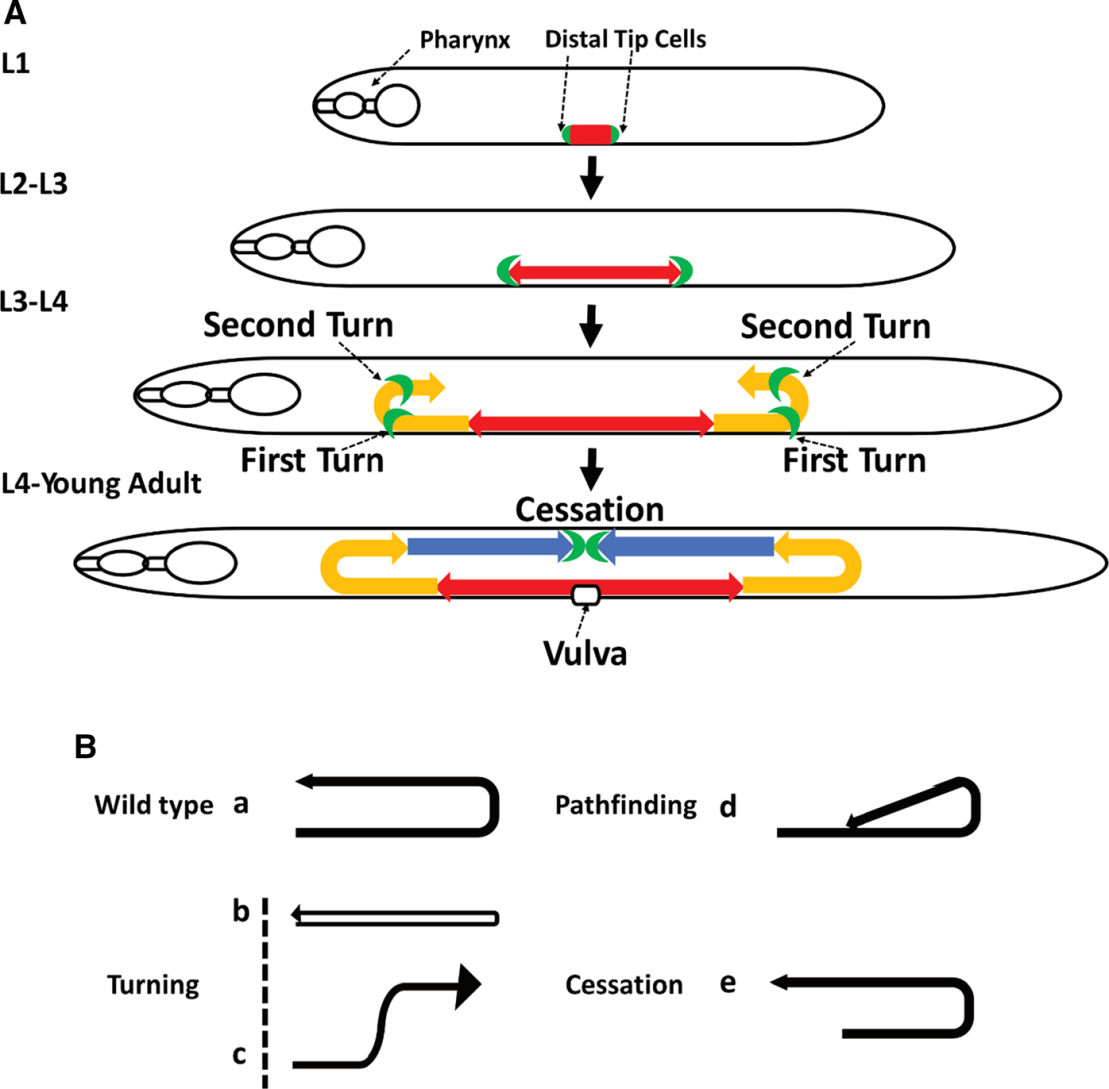
**a** Scheme of the U-shaped migratory path of the distal tip cells (green) that shape the hermaphrodite gonad arms during worm larval development; Phase 1: larval stage 2 (red), phase 2: larval stage 3 (yellow) and phase 3: larval stage 4 (blue). **b** Patterns of DTC migration defects. Arrow shows migration direction. **a** normal U-shaped migratory path, **b** and **c** turning defects (b, ventralized migration; c, wrong turn), **d** pathfinding defects (change in direction),** e** cessation problems (overshoot of migration)

During post-embryonic *C. elegans* development, the morphology of the hermaphroditic gonad is determined. The somatic gonad consists of different tissues, each with specific functions and distinct anatomical features, that are intimately associated with the germ line and have critical roles in development, organization, and function in adult worms. The process determining the gonad takes place during larvae stages L2 to L4 and involves appropriate migration of two specialized leader cells, the so-called distal tip cells (DTCs). At the larval stage L2, the DTCs migrate away from the gonad primordium. At L3, the DTCs turn and migrate to the dorsal side, followed by a U-turn and migration towards the mid-body in the L4 stage (Fig. 2a). Migration ceases opposite to the vulva, and with the two U-shaped gonad arms in place; worms are defined as adults when the vulva completes its development. DTCs are particularly amenable to *in situ* cell migration analysis because (in contrast to most vertebrate tissue) *C. elegans* is transparent. Moreover, the *C. elegans* genome is conserved, which allows translation of functions to other systems, including humans. Dysfunctional DTC migration may result in a range of gonad development defects, such as turning, pathfinding and cessation problems which are easily detected under a microscope (Fig. 2b). Many genes have been identified as crucial for the DTC migration process in worms, including for example Rho GTPases, integrins and metalloproteases (Lundquist et al. 2001; Lee et al. 2005; Wong and Schwarzbauer 2012), but no Cu dependence has been noted.

In this study, taking advantage of the DTC migration *in situ* model, we have characterized *C. elegans* with and without the *cuc-1* gene to reveal a putative role of CUC-1 in the DTC migration process. We found that upon knock-out of the *cuc-1* gene, *C. elegans* animals exhibit defective DTC migration patterns (along with decreased brood size) that can be rescued by wild-type *cuc-1* provided as a transgene. Our results suggest that CUC-1 is a co-regulator of DTC migration during *C. elegans* larvae development.

## Materials and methods

### *C. elegans* worm cultures and strains

**Fig. 3:**
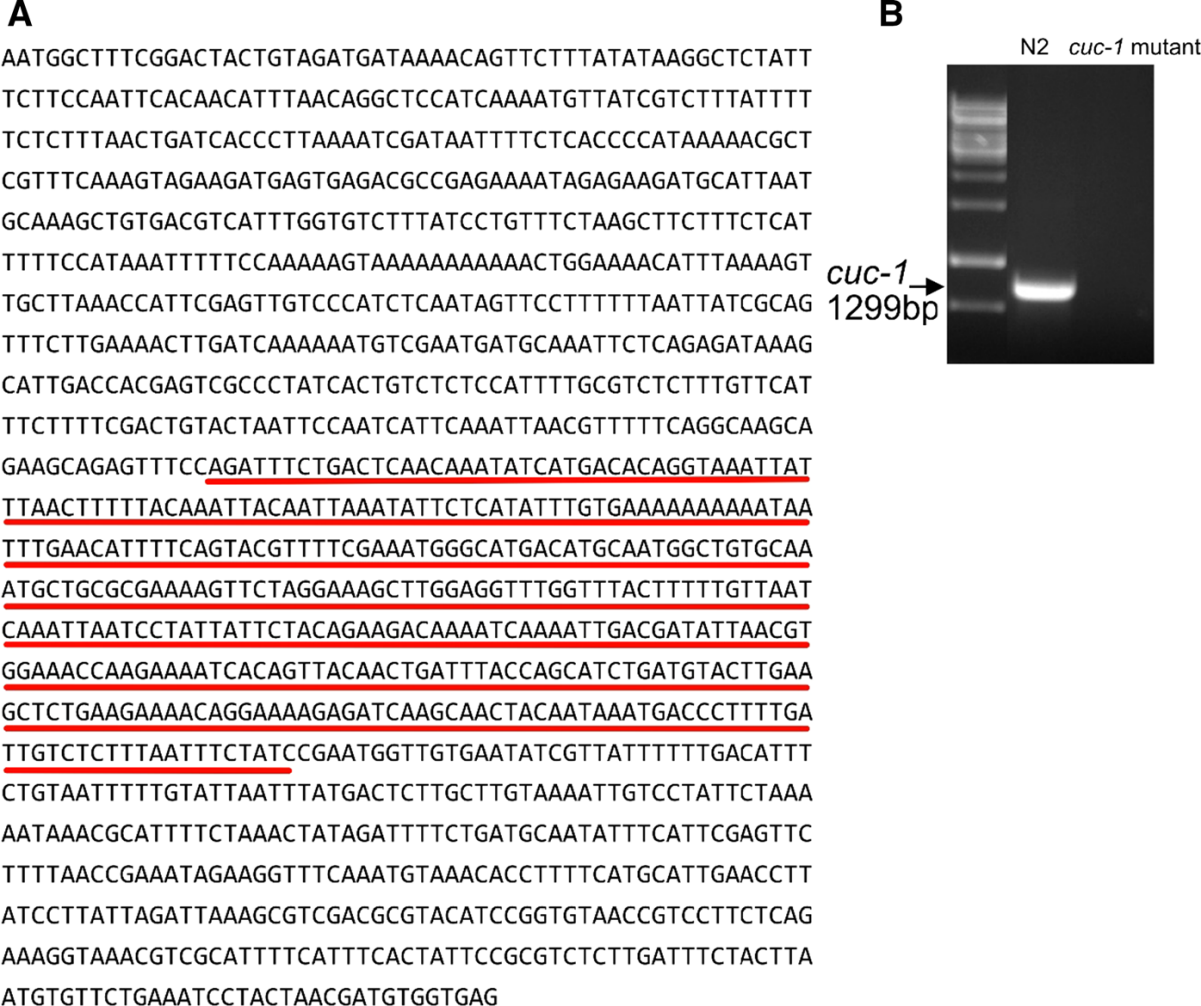
Analysis of *cuc-1* deletion in *C. elegans*. **a** The wild-type *C. elegans cuc-1* DNA sequence. Red underlined part indicates the deletion. **b** PCR data indicate that the *cuc-1* gene is deleted in mutated worms but present in wild-type (N2) worms

*Caenorhabditis elegans* was cultivated at 20 °C on nematode growth medium (NGM) plates with *E. coli* OP50 (worm base ID WBStrain00041969) as the food source. *E. coli* OP50 is an uracil auxotroph whose growth is limited on NGM plates. A limited bacterial lawn is desirable because it allows for easier observation and better mating of the worms. The media, culture and handling of *C. elegans* follow what was described by Brenner (1974). We used two different *C. elegans* strains. The wild-type *C. elegans* strain N2 was provided by the *Caenorhabditis* Genetics Center (funded by the NIH National Center for Research Resources, NCRR), and the PHX1006(*cuc-1(syb1006)/hT2*)) strain was created by Suny Biotech (Fuzhou City, China) using CRISPR/Cas9 to delete *cuc-1* (deletion site shown in Fig. 3a). The deletion of the *cuc-1* gene was confirmed by PCR using the following primers: 5′-AATGGCTTTCGGACTACTGT-3′ and 5′- CTCACCACATCGTTAGTAGG-3′. For Cu complementation, CuCl_2_ was added at a concentration of 50 µM when preparing NGM agar dishes. NGM mixture itself does not contain Cu, however, the *E. coli* OP50 and the yeast extract in the agar may supply Cu to the worms.

### Growth assay

Synchronized worms at L1 stage from both N2 worms (wild type) and *cuc-1* mutant stocks were plated onto NGM plates seeded with OP50. For the growth rate assay, 20–25 worms were mounted and photographed by DIC microscope when worms were grown for 24, 48, 72 and 96 h. Worm length (excluding the thin tail tip) was measured using the ImageJ software (Schindelin et al. 2012).

### Brood size assay

Synchronous L1 worms were plated onto NGM plates with OP50 and after an additional 48 h of growth (to reach L4 stage), 10 worms were singled out onto new plates. The worms were transferred daily during the fertile period and live progeny were counted 3 days after removal of the hermaphrodite.

### Life span assay

Synchronous L4 worms were plated in groups of five onto NGM plates with OP50. 100 worms were picked for both N2 and *cuc-1* mutant strains. The worms were transferred every second day during the fertile period and once a week thereafter. All worms were monitored every day and scored as dead when failing to respond upon several touches on the head with the worm-pick.

### Scoring DTC migration defect phenotypes

Synchronous L1 worms were plated onto NGM plates with OP50, and after an additional 72 h when grown to young adults, hermaphrodites were observed on 2% agar pads using a Zeiss Axio photo microscope equipped with a 60X objective and Nomarski optics (Sulston and Horvitz 1977). 40 worms were analyzed in each experiment, and the experiment was repeated three times. The trajectories of the DTCs were manually deduced from inspections of the gonad arms. Animals were observed with anterior arms on the left and posterior arms on the right side. Both sides were scored.

### Construction of wild-type *cuc-1* rescue plasmid

The *pCUC-1* rescue construct was generated in-house with a Gibson assembly kit (Gibson Assembly® Cloning Kit (New England Biolabs)) using the following primers.

1. 5′- GGAATTCGCCCTTGTCTAGAGTAAGCTATTTTAGAGATTTTTGAC-3′ and 5′- TATCTGCAGAATTCGCCCTTACGTTTACCTTTCTGAGAAG − 3′ to amplify the *cuc-1* gene including 2 kb of upstream regulatory sequence using N2 worm genomic DNA as template.
2. 5′-AAGGGCGAATTCTGCAGATATCCATCACACTGGCGGCCGCTCGA-3′ and 5′- TCTAGACAAGGGCGAATTCCAGCACACTGGCGGCCGTTACTAGTT-3′ as vector primers amplified from *pPAQR-2::GFP* construct (Svensson et al. 2011).

The plasmid construct was confirmed by sequencing using 5′-GTGGCGGCCGCTCTAGAACTAGTAACGGCCGCCAGT-3′ and 5′-TAGATGCATGCTCGAGCGGCCGCCAGTGTGATGGA-3′. The assembled *pCUC-1* plasmid was injected into PHX1006 (*cuc-1*(syb1006)/hT2) worms at 10 ng/µl together with 5 ng/µl *pPD118.33* (*Pmyo-2::GFP*) (Addgene plasmid #1596) and 85 ng/µl pBSKS (Stratagene).

### Statistics

The Student’s *t*-tests were used to determine statistical significance of our measures. All experiments were repeated at least three times. Asterisks presented in the figures indicate various degrees of significance, as follows: *p < 0.05, **p < 0.01, ***p < 0.001.

## Results

**Fig. 4:**
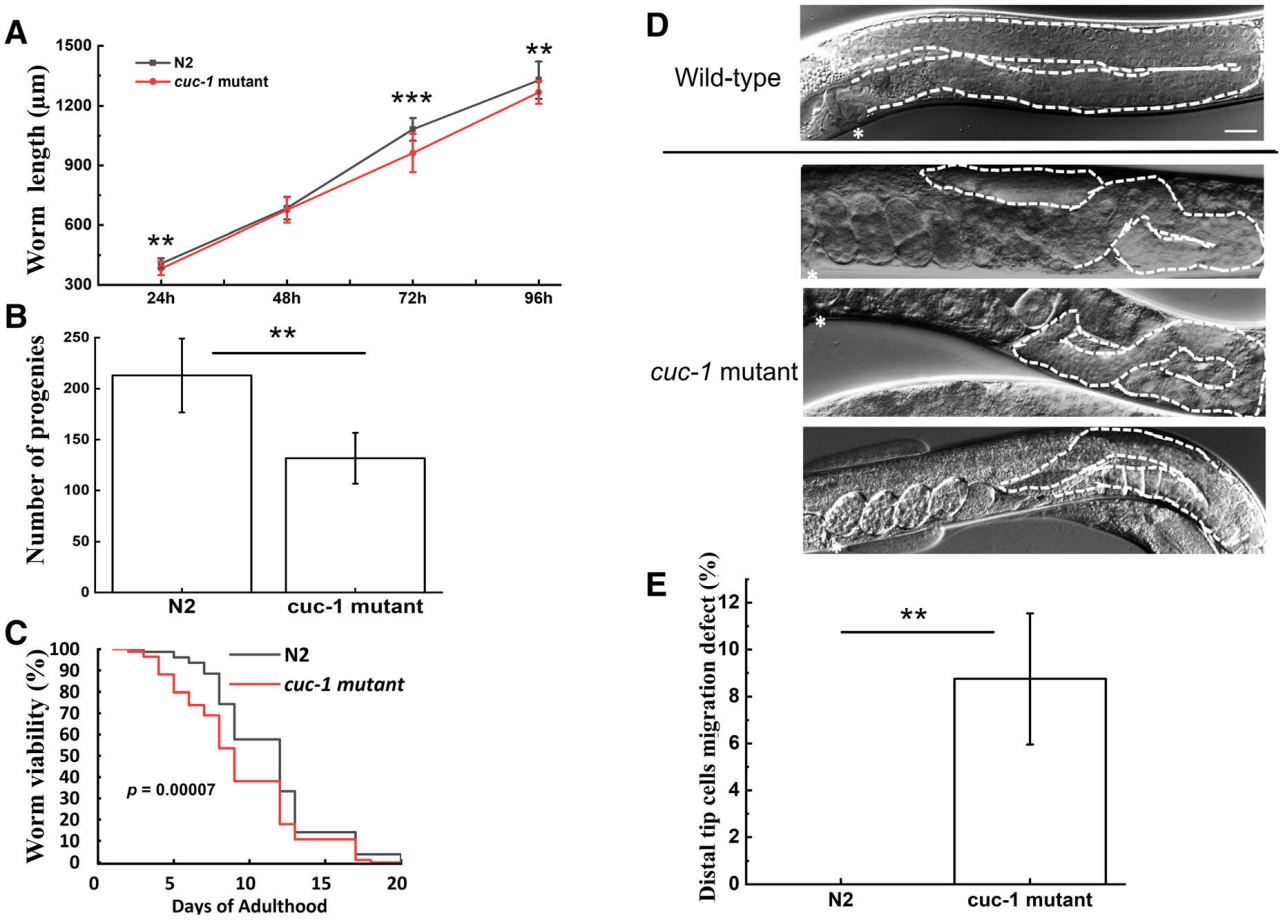
Characterization of *cuc-1* mutant versus wild-type (N2) *C. elegans* at 24, 48, 72 and 96 h of incubation post L1 stage (condition with no supplemental Cu). **a** Growth assay (n_group_=25). **b** Brood size assay (n_group_=10). **c** Life span assay (n_group_=100). **d** Examples of DTC migration defects found in *cuc-1* mutant *C. elegans*. Asterisks indicate vulva. White dashed lines indicate gonad shapes. The scale bar in wild-type image represents 50 µm for all images. **e** Quantification of DTC migration defects in *cuc-1* mutant *C. elegans* (n_group_=40; three independent groups analyzed). Statistical significance determined using the Student´s *t*-test with * for *p* < 0.05, ** for *p* < 0.01, *** for *p* < 0.001

*C. elegans* worms with the *cuc-1* gene deleted (*cuc-1* mutant) using CRISPR-Cas9 technology (Fig. 3a) were tested with PCR (Fig. 3b) to assure successful gene deletion. Importantly, the deletion was not lethal to worms. To determine the effects of the *cuc-1* mutation on worm properties, *C. elegans* worms were grown on NGM plates fed with the *E. coli* strain OP50 over 20 days. The worm growth assay showed that *cuc-1* mutant worms exhibited wild-type length during development except for the L4 to young adult stage, where a ~ 10% reduction in length was noted (Fig. 4a). Importantly, the *cuc-1* mutant displayed a 50% reduction in brood size compared to wild-type worms (Fig. 4b), and the *cuc-1* mutant also showed a somewhat shorter life span (*p* = 0.00007) than wild-type worms (Fig. 4c).

Next, we analyzed the DTC migration patterns by scoring wild-type and *cuc-1* mutant worms at the young adult stage. Upon analysis of defects, we found that almost 10% of *cuc-1* mutants had DTC migration defects (Fig. 4 d,e), whereas no such defects were observed in wild-type worms. Table 1 reports the types of DTCs migration defects (Fig. 2b) observed, revealing that most defects (in more than half of defective *cuc-1* mutant worms) were classified as pathfinding defects.

**Table 1:** Classification of DTC migration defects (see Fig. 2b) found in *cuc-1* mutant worms at the following two conditions: the normal growth condition and the supplemented condition with 50 µM Cu

Condition	Classification of DTC migration defects (%)			
	Turning	Pathfinding	Cessation	Other
Normal	34 ± 10	50 ± 6	12 ± 11	4 ± 8
Added Cu	39 ± 16	51 ± 20	5 ± 8	5 ± 8

**Fig. 5:**
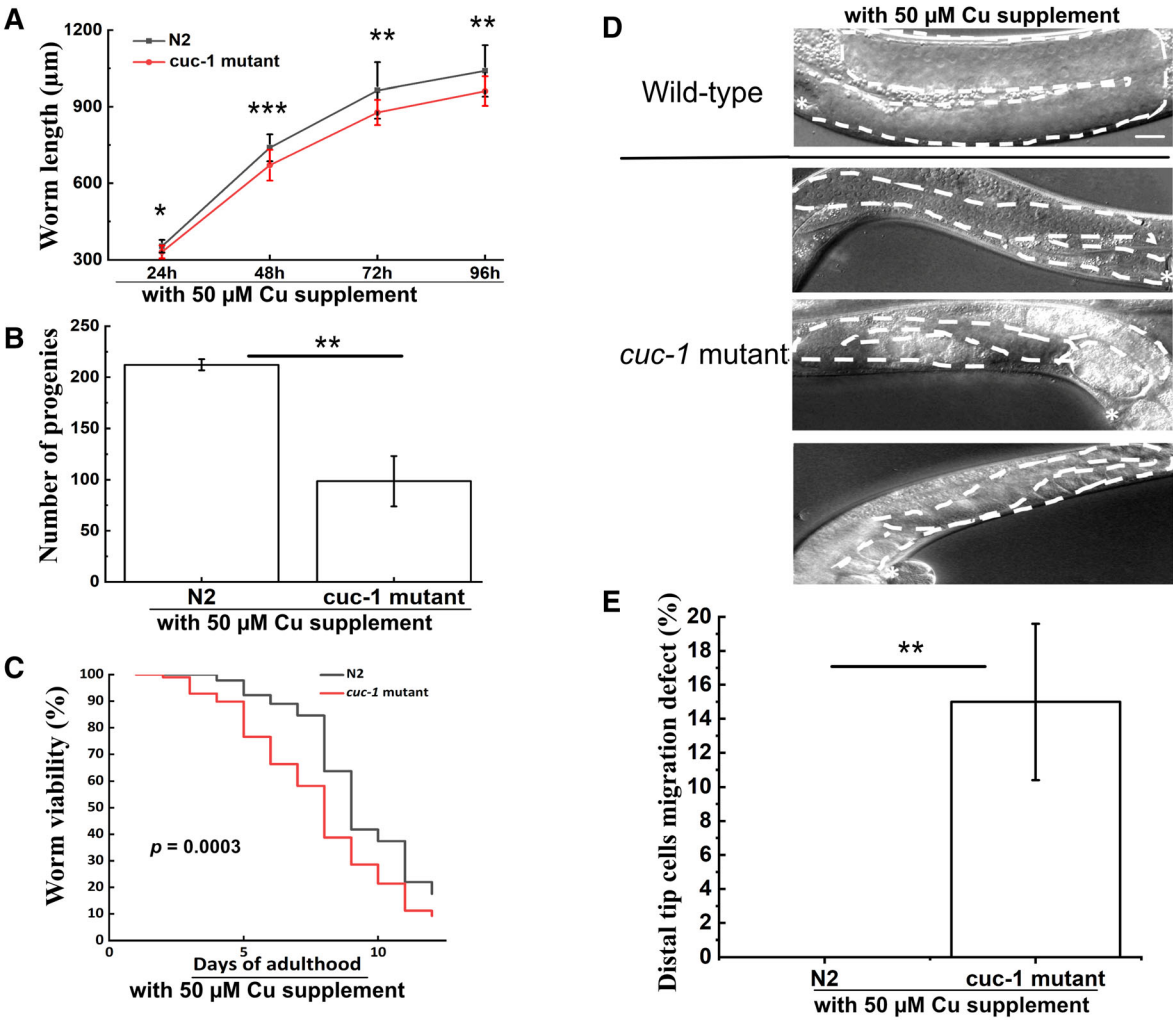
Characterization of *cuc-1* mutant versus wild-type (N2) *C. elegans* at 24, 48, 72 h incubation post L1 stage (condition with 50 µM supplemental Cu). **a** Growth assay (n_group_=25). **b** Brood size assay (n_group_=10). **c** Life span assay (n_group_=100). **d** Examples of DTC migration defects found in *cuc-1* mutant *C. elegans*. Asterisks indicate vulva. White dashed lines indicate gonad shapes. The scale bar in wild-type image represents 50 µm for all images. **e** Quantification of DTC migration defects for *cuc-1* mutant *C. elegans*. (n_group_=40; three independent groups analyzed). Statistical significance determined using the Student´s *t*-test with * for *p* < 0.05, ** for *p* < 0.01, *** for *p* < 0.001

**Fig. 6:**
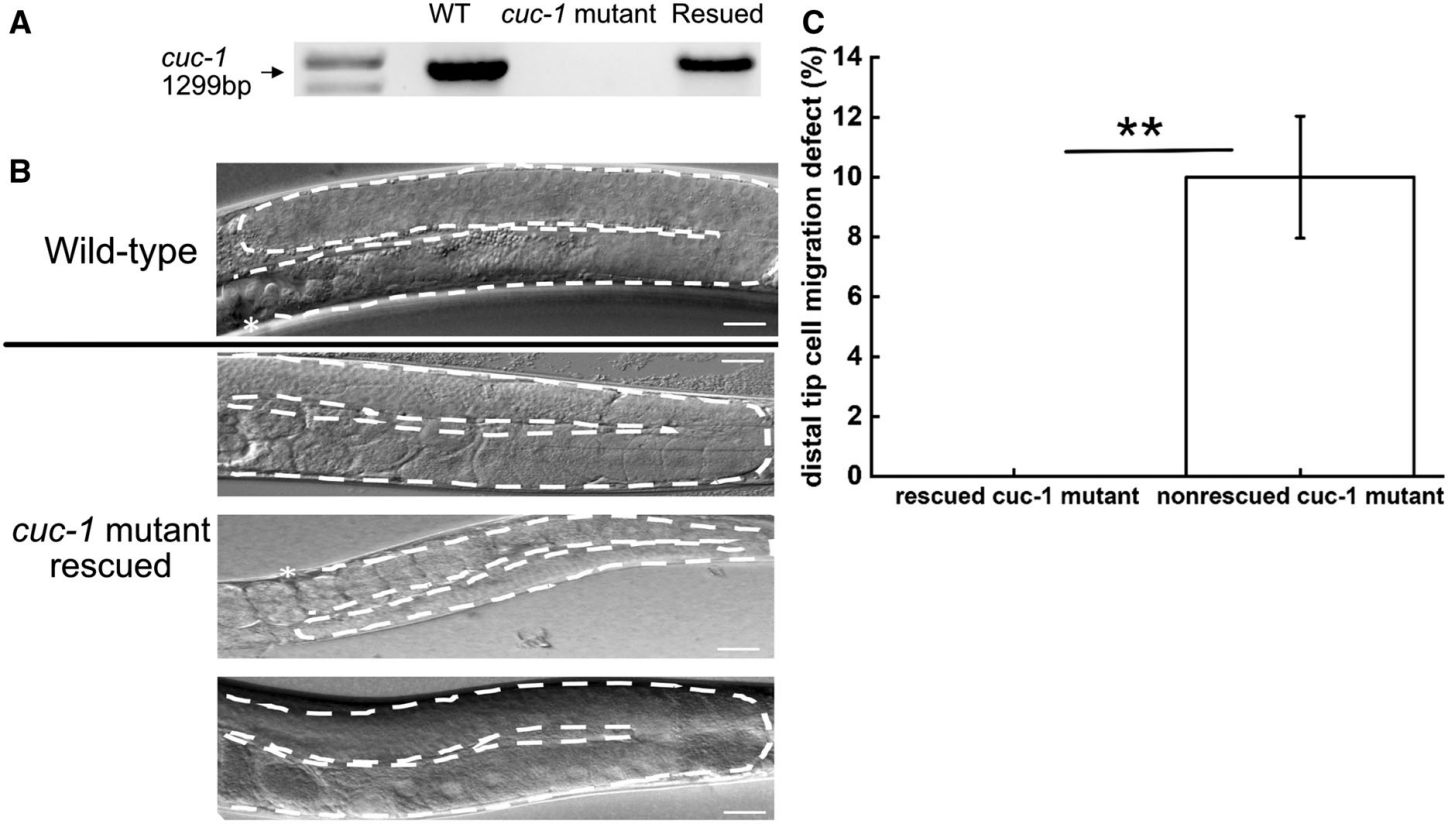
**a** PCR data show the *cuc-1* gene to be successfully inserted into *cuc-1* mutant worms upon transgene plasmid microinjection. **b** Examples of DTC migration in rescued *cuc-1* mutant *(C) elegans* (n_group_=40; three independent groups analyzed). Asterisks indicate vulva. White dashed lines indicate gonad shapes. No DTC migration defects were detected. The scale bar represents 50 µm. **c** Quantification of DTC migration defects for rescued *cuc-1* mutant and non-rescued mutant *C. elegans* worms analyzed in parallel. Statistical significance determined using the Student´s *t*-test with * for *p* < 0.05, ** for *p* < 0.01, *** for *p* < 0.001

The data at the ‘normal’ condition include 25 defective worms out of the 240 analyzed worms (data from experiments in Fig. 4 and non-rescued mutant worms in Fig. 6 merged). The data at the ‘added Cu’ condition include 18 defective worms out of 120 analyzed worms (data from experiments in Fig. 5). ‘Other’ refer to DTC migration defects not possible to classify into the other three categories (turning, pathfinding and cessation problems). Standard deviations for the values are calculated from 4 to 5 replicates. We note that only for turning and pathfinding defects, the data are of significance

The normal growth condition for the worms (NGM petri plates with *E. coli* OP50 food) does not include added Cu, although the *E. coli* and the yeast extract in the agar are sources of Cu and thus the worms have access to some Cu (but exact concentrations are not known). Previous studies have shown that removal of all Cu by addition of chelation compounds, or addition of excessive levels of Cu to the growth media, diminish growth of *C. elegans*, but for up to 100 µM of Cu supplemented in the NGM agar, there are no growth defects noted in worms (Chun et al. 2017). Therefore, to test whether *cuc-1* mutants are sensitive to the level of Cu, we compared the phenotypes of wild-type and *cuc-1* mutants grown on normal plates or NGM plates supplemented with 50 µM Cu. Thus, although both conditions will contain Cu, the latter should give the worms access to more Cu.

At the 50 µM Cu supplemented condition, we found again a 50% reduction of brood size and shorter worm lengths at the L4 to adult stages for the *cuc-1* mutant worms; in addition, for this condition we also found shorter worm lengths at L3 to L4 stages for the *cuc-1* mutant worms (Fig. 5a, b). The life span assay showed again a somewhat reduced life span for *cuc-1* mutant worms as compared to wild-type worms (Fig. 5c). In the Cu-supplemented condition, 15% of *cuc-1* mutant worms exhibited DTC migration defects (Fig. 5d, e) and again pathfinding defects dominated (Table 1). Importantly, DTCs migration in wild-type worms remained unaffected at the Cu-supplemented condition. The similar outcomes for the two different Cu conditions imply that the observed defects due to *cuc-1* deletion is not sensitive to variation in external Cu supply. We note that we have not assessed Cu content inside the worms (e.g., in the DTCs) which, due to regulation of cellular Cu uptake and transport in worms (Wakabayashi et al. 1998; Chun et al. 2017), may be rather similar at the two conditions.

To prove that the observed DTC migration defects are due to absence of the CUC-1 protein, and not due to indirect effects of the gene deletion, we injected a plasmid containing the wild-type *cuc-1* gene (named *pCUC-1*) into *cuc-1* mutant worms. First, *pCUC-1* was injected into the gonad syncytium of *cuc-1* mutant worms to establish a transgenic line. Transgenic worms in subsequent generations were verified by PCR (Fig. 6a). Scoring of ‘rescued’ worms at the young adult stage showed no DTC migration defects (Fig. 6b), whereas, as before, around 10% of the non-transgenic *cuc-1* mutant worms investigated in parallel exhibited DTC migration defects (Fig. 6c). Thus, by adding back the *cuc-1* gene, *cuc-1* mutant worms are rescued with respect to migration of the DTCs. This demonstrates that the migration defects observed in *cuc-1* mutants are due to lack of CUC-1 protein.

## Discussion

Gonad morphogenesis in *C. elegans* is dependent on specific migration patterns of DTCs and, for many reasons, is an excellent system to dissect the molecular basis of developmental cell migration (Lee et al. 2005). Several proteins and pathways have been related to the distinct steps of DTC migration that eventually results in two U-shaped gonad arms (Lundquist et al. 2001; Lee et al. 2005). Because the human Cu chaperone Atox1 has been found to facilitate cancer cell migration (Blockhuys et al. 2020) and play an apparent role in pre-implantation mouse embryo development (Celauro et al. 2017), we here set out to test the role of the *C. elegans* Atox1 homolog, CUC-1, in DTC migration.

It has been reported that CUC-1, together with CUA-1 (which is the *C. elegans* homolog to human ATP7A/B), function in Cu transport as in humans, but with tissue-specific expression (Wakabayashi et al. 1998; Chun et al. 2017). Here, we investigated *C. elegans* with the *cuc-1* gene removed using CRISPR-Cas9 technology. In similarity to a study in which the *cuc-1* gene was silenced using RNAi (Chun et al. 2017), we found the mutated worms to display reduced brood size. Because the deletion of the gene did not affect worm growth and life span much, we were able to probe migration of DTCs during larval development as a function of CUC-1 presence.

We found that whereas wild-type worms had no DTC migration defects at the two conditions studied, approximately 10% (normal growth condition) and 15% (50 µM Cu supplemented to the plates) of *cuc-1* mutant worms showed DTC migration defects. When the defects were classified (at both conditions), we found pathfinding problems to be most common followed by turning problems. However, our classification gives only rough estimates as, in some cases, more than one problem may be present, and the total number of defective worms analyzed are small. The results for CUC-1 deletion should be placed in relation to similar studies investigating the roles of other gene products in DTC migration. Reporting migration defects as a percentage of worms with defects is a common way to quantify dysfunction due to gene mutations. In other studies, the percentage of worms with DTC migration defects range from around 10–20% defective worms to much higher percentages, depending on the gene deleted. Rho GTPases such as CED-10 and MIG-2 are known to play key roles in DTC migration (Lundquist et al. 2001; Lee et al. 2005; Wong and Schwarzbauer 2012) and are regulated by many additional proteins (Demarco and Lundquist 2010; Tannoury et al. 2010). In similarity to the detected magnitudes of defects here for *cuc-1* deletion, mutation in the *mig-2* and *ced-10* genes resulted in DTC migration defects (with pathfinding problems highlighted) in 27% and 12% of worms, respectively (Lundquist et al. 2001). Nonetheless, due to the low percentage of worms affected by *cuc-1* deletion, it may be speculated that there is functional redundancy such that other proteins can substitute (in part) for CUC-1.

We note that the observation for CUC-1 is not a general result of removing a Cu-binding protein from the organism, as it was reported that gene knockdown of the Cu-binding protein CUTC-1 (an ortholog of human CUTC) in *C. elegans* did not affect brood size, worm length, or DTC migration at added Cu concentrations up to 100 µM (Calafato et al. 2008). Instead, CUTC-1 was concluded to protect worms from toxic effects of excess Cu (above 100 µM).

Our findings demonstrate that the Atox1 homolog in worms, CUC-1, plays a role in promoting correct DTC migration during *C. elegans* development. Thus, CUC-1 should be considered a new modulator of the extensive network of proteins that facilitate DTCs migration in worms. CUC-1 may play Cu-dependent or Cu-independent roles in DTC migration. We speculate that there may be unknown Cu dependencies for other proteins in these processes to which CUC-1 would deliver Cu. Speculatively, Cu-loaded CUC-1 may help mediate oxidation/reduction processes that regulate Rho GTPases implicated in DTC migration, such as Rac, Rho, and Cdc-42. In human cells, Atox1 was found to mediate cancer cell migration by (via Cu transport) promoting extracellular, Cu-dependent, collagen-remodeling lysyl oxidase (LOX) activity. *C. elegans* worms do not have LOX proteins but instead depend on other proteins that regulate the extracellular matrix. No Cu dependence for those proteins have been reported but, nonetheless, many are oxidases, peroxidases and thioredoxin-like proteins (Myllyharju and Kivirikko 2004) that may turn out to depend on Cu. There are also possible Cu-independent mechanisms responsible for the observations: CUC-1 may transmit signals via protein-protein interactions that may be dependent on redox status of CUC-1’s two cysteine residues (in the Cu-binding loop). Such redox properties have been reported for Atox1 (Hatori and Lutsenko 2013) and may be conserved in CUC-1. Taken together, Atox1 and its homologs (e.g., CUC-1) appear to have functions, beyond that of pure cytoplasmic Cu transport, that aid in cell migration during organism development.

## Data Availability

All data and material are available upon request from the corresponding author.
